# Supplementary material for: Diet-driven differential response of Akkermansia muciniphila modulates pathogen susceptibility
Source: Mol Syst Biol. 2024 May 14;20(6):2. doi: 10.1038/s44320-024-00036-7 (PMC11148096; doi:10.1038/s44320-024-00036-7)
Supplement: Supplementary file 1 — Table EV1 [file 44320_2024_36_MOESM1_ESM.pdf]

Extended View

Diet-driven differential response of *Akkermansia muciniphila* modulates pathogen susceptibility

Mathis Wolter[1,2], Erica T. Grant[1,2], Marie Boudaud[1], Nicholas A. Pudlo[3], Gabriel V. Pereira[3], Kathryn A. Eaton[3], Eric C. Martens[3] and Mahesh S. Desai[1,4,*]

[1]Department of Infection and Immunity, Luxembourg Institute of Health, Esch-sur-Alzette, Luxembourg

[2]Faculty of Science, Technology and Medicine, University of Luxembourg, Esch-sur-Alzette, Luxembourg

[3]Department of Microbiology and Immunology, University of Michigan Medical School, Ann Arbor, Michigan, USA

[4]Odense Research Center for Anaphylaxis, Department of Dermatology and Allergy Center, Odense University Hospital, University of Southern Denmark, Odense, Denmark

[*]Corresponding author, email: mahesh.desai@lih.lu

Table EV1. Genome accession details for metatranscriptome analyses in Salmon.			
**Organism Scientific Name**	**Organism Qualifier**	**Assembly Accession**	**Submission Date**
Akkermansia muciniphila	strain: ATCC BAA-835	GCF_000020225.1	5/5/2008
Bacteroides caccae	strain: ATCC 43185	GCF_025146315.1	9/12/2022
Barnesiella intestinihominis	strain: YIT 11860	GCF_000296465.1	9/17/2012
Bacteroides ovatus	strain: ATCC 8483	GCF_001314995.1	10/15/2015
Bacteroides uniformis	strain: ATCC 8492	GCF_025147485.1	9/12/2022
Bacteroides thetaiotaomicron	strain: DSM 2079	GCF_014131755.1	8/10/2020
[Clostridium] symbiosum	strain: ATCC 14940	GCF_000466485.1	9/12/2013
Collinsella aerofaciens	strain: JCM 10188	GCF_010509075.1	2/13/2020
Faecalibacterium prausnitzii	strain: A2165	GCF_002734145.1	10/27/2017
Roseburia intestinalis	strain: L1-82	GCF_900537995.1	2/27/2019
Desulfovibrio piger	isolate: FI11049	GCF_900116045.1	11/12/2016
Marvinbryantia formatexigens	strain: DSM 14469	GCF_025148285.1	9/12/2022
Escherichia coli	strain: HS	GCF_000017765.1	9/10/2007
[Eubacterium] rectale	strain: VPI 0990	GCA_022453685.1	3/3/2022
Citrobacter rodentium	strain: ATCC 51459	GCF_000835925.1	2/10/2015
