# Supplementary material for: Diet-driven differential response of Akkermansia muciniphila modulates pathogen susceptibility
Source: Mol Syst Biol. 2024 May 14;20(6):2. doi: 10.1038/s44320-024-00036-7 (PMC11148096; doi:10.1038/s44320-024-00036-7)
Supplement: Supplementary file 14 — Expanded View Figures [file 44320_2024_36_MOESM14_ESM.pdf]

Expanded View Figures

Figure EV1. Synthetic microbiota profiles through colonization, feeding period, and *Citrobacter rodentium* infection.

Relative abundances of 14SM, 13SM, 12SM, 11SM, and 10SM bacteria among mice fed a fiber-rich (FR, left panels) or fiber-free (FF, right panels) diet. Abundances were calculated from 16S rRNA gene sequencing results after initial colonization (day 6–8, $n = 0$–9), prior to diet change (day 10–13, $n = 4$–13), during the 40-day feeding period (day 15–53, $n = 3$–14), or during *Citrobacter rodentium* infection (day 54–64, $n = 4$–13). The number of sampling points varies by SM, with 31–39 for 14SM, 4 for 13SM, 33–34 for 12SM, 15–16 for 11SM, and 16–18 for 10SM.

© The Author(s)

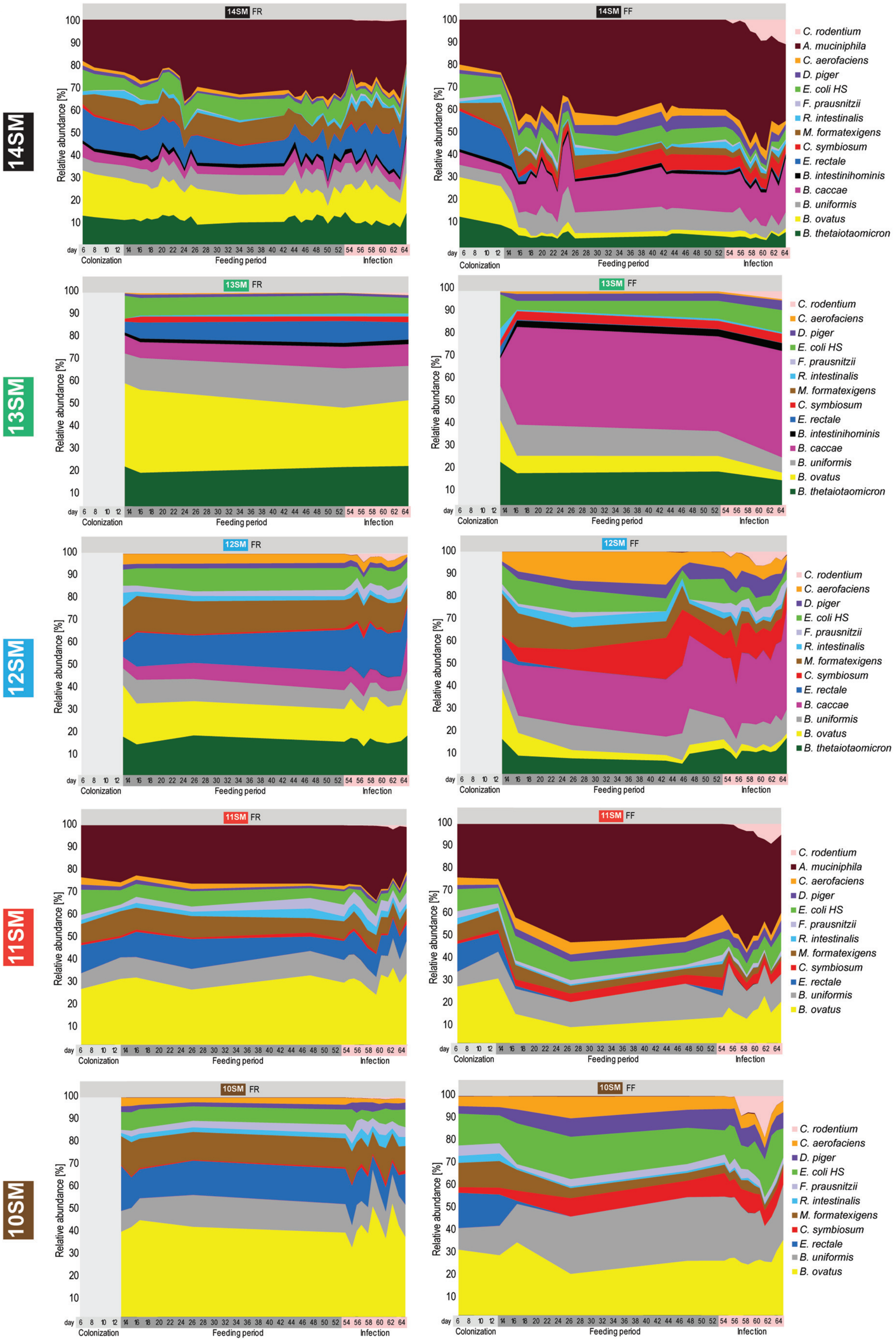
14SM
14SM FR
14SM FF
13SM
13SM FR
13SM FF
12SM
12SM FR
12SM FF
11SM
11SM FR
11SM FF
10SM
10SM FR
10SM FF
Relative abundance [%]
day
Colonization
Feeding period
Infection
C. rodentium
A. muciniphila
C. aerofaciens
D. piger
E. coli HS
F. prausnitzii
R. intestinalis
M. formatexigens
C. symbiosum
E. rectale
B. intestinihominis
B. caccae
B. uniformis
B. ovatus
B. thetaiotaomicron

 © The Author(s)

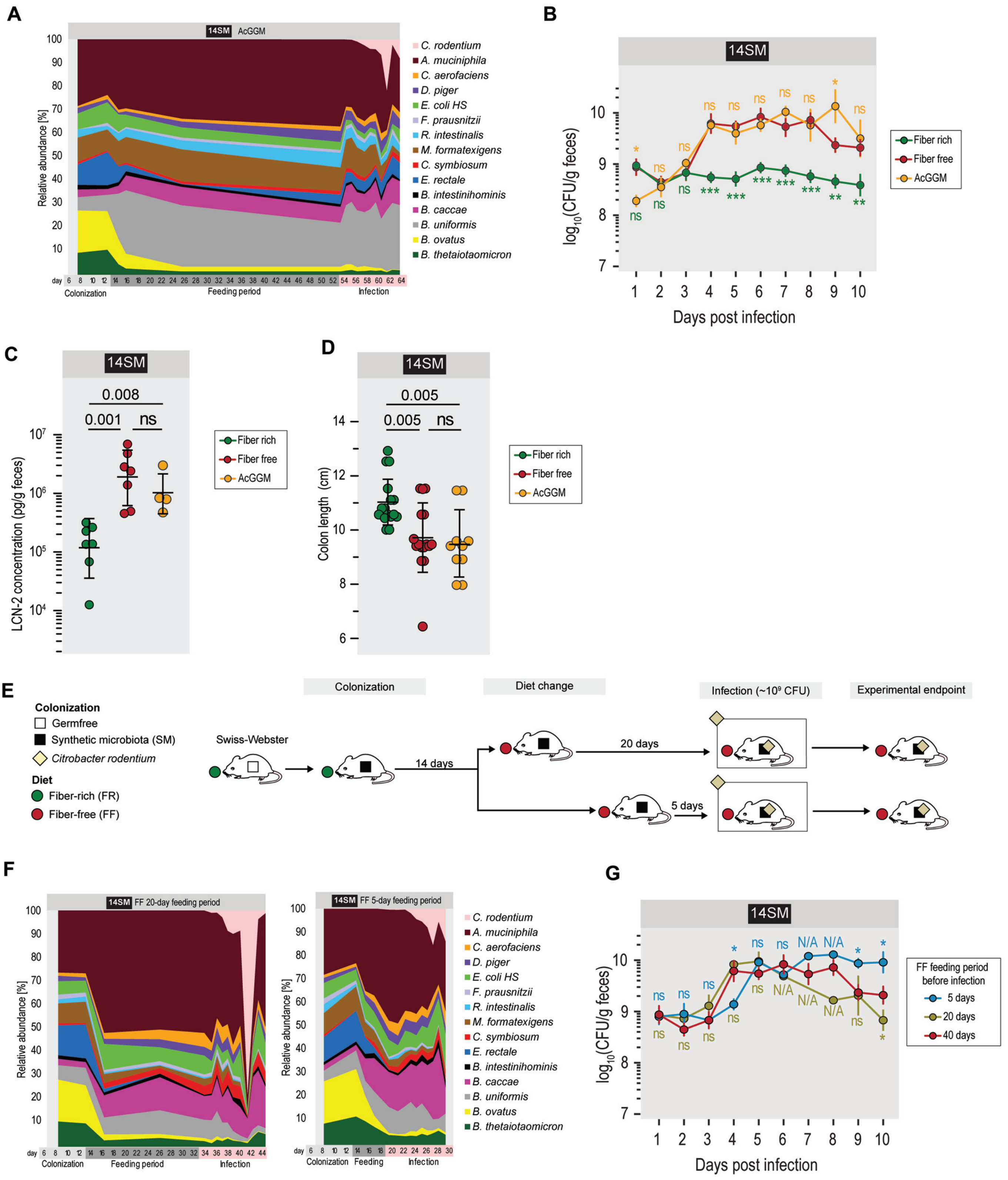
A
14SM AcGGM
Relative abundance [%]
C. rodentium
A. muciniphila
C. aerofaciens
D. piger
E. coli HS
F. prausnitzii
R. intestinalis
M. formatexigens
C. symbiosum
E. rectale
B. intestinihominis
B. caccae
B. uniformis
B. ovatus
B. thetaiotaomicron
Colonization
Feeding period
Infection
B
14SM
log10(CFU/g feces)
Days post infection
Fiber rich
Fiber free
AcGGM
C
14SM
LCN-2 concentration (pg/g feces)
D
14SM
Colon length (cm)
E
Colonization
Germfree
Synthetic microbiota (SM)
Citrobacter rodentium
Diet
Fiber-rich (FR)
Fiber-free (FF)
Swiss-Webster
Diet change
14 days
20 days
5 days
Infection (~10⁹ CFU)
Experimental endpoint
F
14SM FF 20-day feeding period
14SM FF 5-day feeding period
Feeding
G
14SM
FF feeding period before infection
5 days
20 days
40 days

◀ **Figure EV2. Acetylated galactoglucomannan supplementation is insufficient to rescue the resistant phenotype of the FR-fed mice, while short-term feeding of the FF diet is sufficient to develop a susceptible phenotype.**

(**A**) Relative abundances of 14SM bacteria among mice fed a fiber-free (FF) diet supplemented with 7.5% acetylated galactoglucomannan (AcGGM). Abundances were calculated from 16S rRNA gene sequencing results after initial colonization (day 8), prior to diet change (day 13), during the feeding period (day 15–54), or during *C. rodentium* infection. Ten sampling points, $n = 1$–5 per sampling point. (**B**) Fecal *C. rodentium* load of 14SM FR-, FF- and AcGGM-fed Swiss Webster mice during the 10 days of infection ($n = 5$–11 per group following the exclusion of three outliers). Error bars show SEM and the curves the arithmetic mean; one-way ANOVA with adjustment for multiple comparisons between FF and AcGGM (top significance labels, yellow text) or between FF and FR (bottom significance labels, green text) group using the Benjamini–Hochberg method. ns, non-significant; *, adjusted $p < 0.1$; **, adjusted $p < 0.01$; ***, adjusted $p < 0.001$. (**C**) Fecal LCN-2 concentration of infected 14SM FR-, FF-, and AcGGM-fed mice on the final day of their infection ($n = 4$–7 per group, no outliers). Error bars show SD and the center of the arithmetic mean; one-way ANOVA with adjustment for multiple comparisons using the Benjamini–Hochberg method. ns non-significant. (**D**) Colon length of infected 14SM FR-, FF-, and AcGGM-fed mice on the final day of their infection ($n = 10$–16 per group, no outliers). Error bars show SD and the center of the arithmetic mean; one-way ANOVA with adjustment for multiple comparisons using the Benjamini–Hochberg method. ns non-significant. (**E**) Experimental timeline for short-term feeding experiment. Age-matched germ-free Swiss Webster mice were gavaged with one of the different synthetic gut microbiota (SM) on 3 consecutive days. These mice were maintained for at least 14 days on the fiber-rich (FR) diet, after which mice were switched to a fiber-free (FF) for either 20 days (FF, $n = 3$) or 5 days (FF, $n = 4$) before they were infected with *Citrobacter rodentium*. After infection, mice were closely observed for up to 10 days. (**F**) Relative abundances of 14SM bacteria among mice fed a fiber-free diet for 20 (left) and 5 (right) days prior to infection. Abundances were calculated from 16S rRNA gene sequencing results, and the number of sampling points varies by feeding regimen, with 16 for the FF diet with a 20-day feeding period and 10 for the FF diet with a 5-day feeding period, $n = 1$–4 mice per sampling point. (**G**) Fecal *C. rodentium* load of 14SM mice fed the FF diet for 5, 20, or 40 days before infection. 5-day feeding $n = 4$, 20-day feeding $n = 3$, 40-day feeding $n = 12$. Some days it was impossible to recover fecal pellets from certain mice. Error bars show SEM and the curves the arithmetic mean; one-way ANOVA with adjustment for multiple comparisons between 5-day feeding and 40-day feeding (top significance labels, blue text) groups or between 20-day feeding and 40-day feeding (bottom significance labels, olive green text) groups using the Benjamini–Hochberg method. N/A, no statistic available as $n = 1$ for one group; ns non-significant; *, adjusted $p < 0.1$; **, adjusted $p < 0.01$; ***, adjusted $p < 0.001$.

© The Author(s)

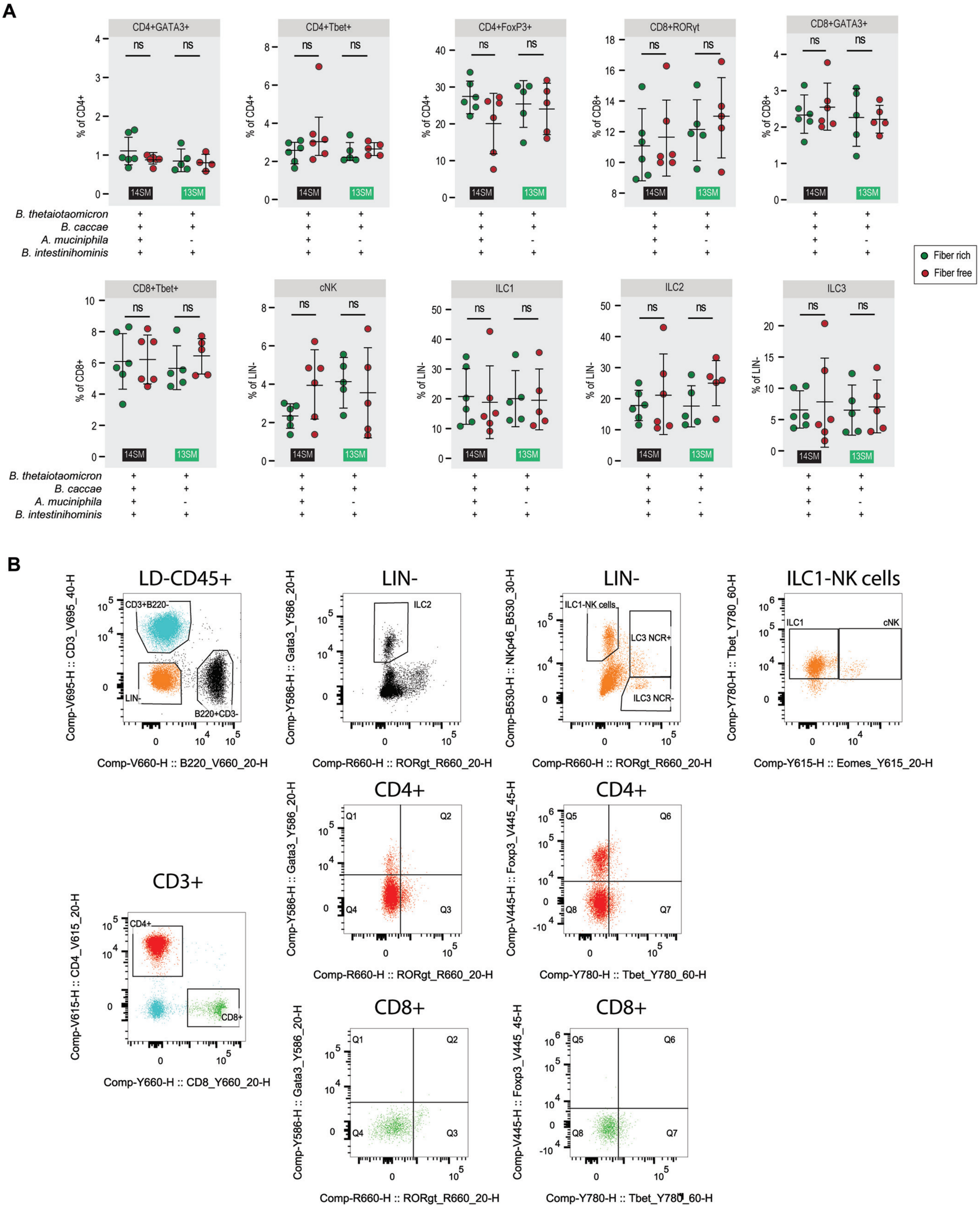
A
CD4+GATA3+
CD4+Tbet+
CD4+FoxP3+
CD8+RORγt
CD8+GATA3+
CD8+Tbet+
cNK
ILC1
ILC2
ILC3
% of CD4+
% of CD8+
% of LIN-
ns
14SM
13SM
B. thetaiotaomicron
B. caccae
A. muciniphila
B. intestinihominis
Fiber rich
Fiber free
B
LD-CD45+
LIN-
ILC1-NK cells
CD3+
CD4+
CD8+
CD3+B220-
LIN-
B220+CD3-
ILC2
LC3 NCR+
ILC3 NCR-
ILC1
cNK
Comp-V695-H :: CD3_V695_40-H
Comp-V660-H :: B220_V660_20-H
Comp-Y586-H :: Gata3_Y586_20-H
Comp-R660-H :: RORgt_R660_20-H
Comp-B530-H :: NKp46_B530_30-H
Comp-Y780-H :: Tbet_Y780_60-H
Comp-Y615-H :: Eomes_Y615_20-H
Comp-V445-H :: Foxp3_V445_45-H
Comp-V615-H :: CD4_V615_20-H
Comp-Y660-H :: CD8_Y660_20-H

◀ **Figure EV3. Host immune cell populations remain largely unchanged between dietary groups after infection with *C. rodentium.***

(**A**) Non-significantly altered immune cell population as a percent of the parent population among mice at 3 DPI, as determined by fluorescence-activated cell sorting (FACS). Population percentages were analyzed by two-way ANOVA (main effects only) with adjustment for multiple comparisons between SMs of the same diet (shown) and between diets of the same SM (see Source Data) using the Benjamini–Hochberg method ($n = 4$–6 per group, following exclusion of one outlier in 13SM FF CD + GATA3+ group). Error bars show SD and the center of the arithmetic mean; ns non-significant (adjusted $p > 0.1$). (**B**) Gating strategy for FACS analysis.

© The Author(s)

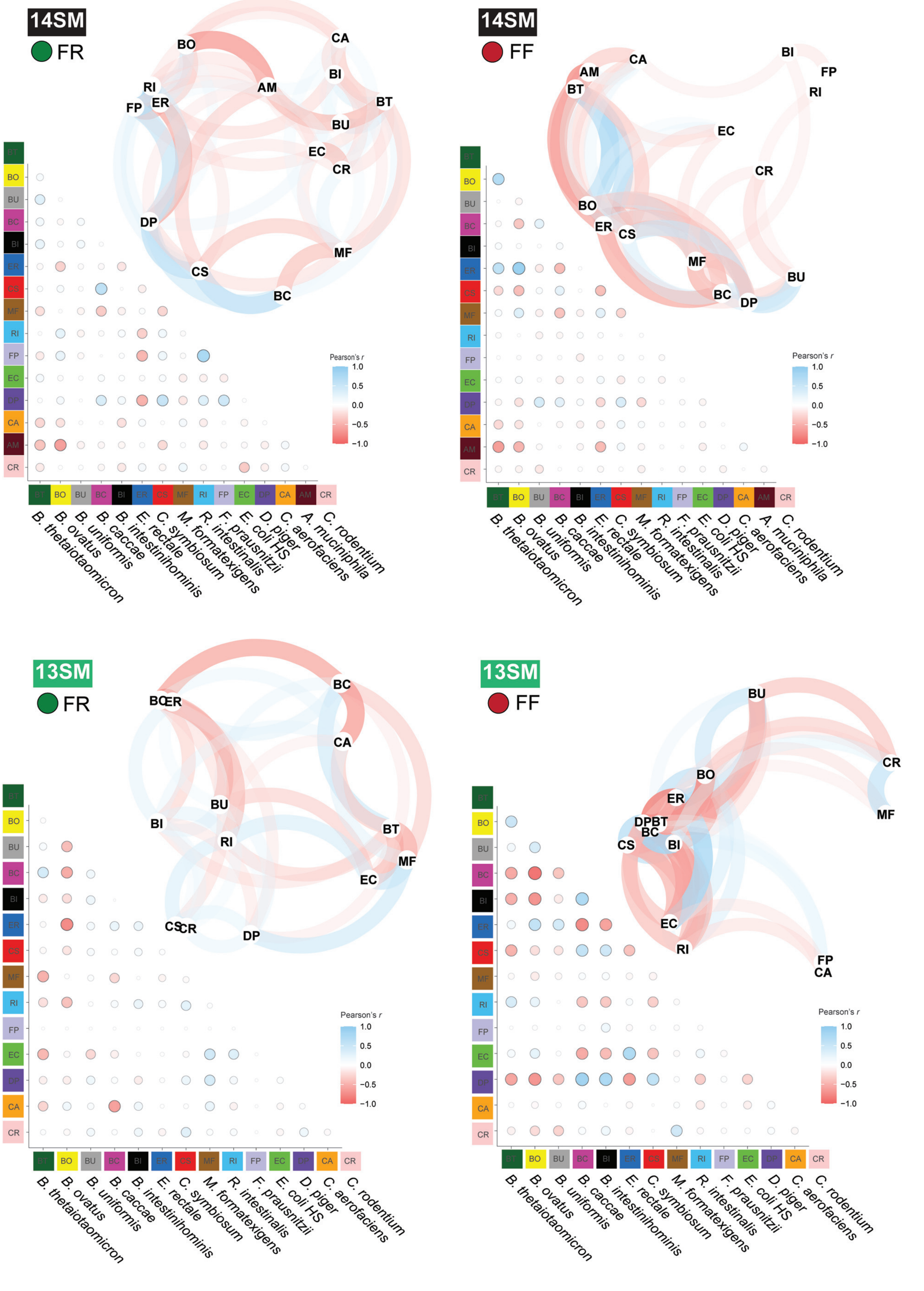
14SM
FR
BO
CA
BI
RI
AM
FP
ER
BT
BU
EC
CR
DP
MF
CS
BC
Pearson's r
1.0
0.5
0.0
−0.5
−1.0
14SM
FF
AM
CA
BI
FP
BT
RI
EC
CR
BO
ER
CS
MF
BU
BC
DP
BT
BO
BU
BC
BI
ER
CS
MF
RI
FP
EC
DP
CA
AM
CR
B. thetaiotaomicron
B. ovatus
B. uniformis
B. caccae
B. intestinihominis
E. rectale
C. symbiosum
M. formatexigens
R. intestinalis
F. prausnitzii
E. coli HS
D. piger
C. aerofaciens
A. muciniphila
C. rodentium
13SM
FR
BC
BO
ER
CA
BU
BI
BT
RI
MF
EC
CS
CR
DP
13SM
FF
BU
CR
BO
ER
MF
DP
BT
BC
CS
BI
EC
RI
FP
CA

Figure EV4. Distinct effects of diet and community composition on bacterial co-abundance correlations.

Correlation matrices and networks of relative abundances in Dataset EV1 across all timepoints (14 FR, $n = 177$; 14SM FF, $n = 200$; 13SM FR, $n = 20$; 13SM FF, $n = 19$). In the matrix (lower left), all correlations are shown. Correlations were calculated using the Pearson method. In the network plot (upper right), only correlations with $|r| > 0.2$ are shown.

© The Author(s)

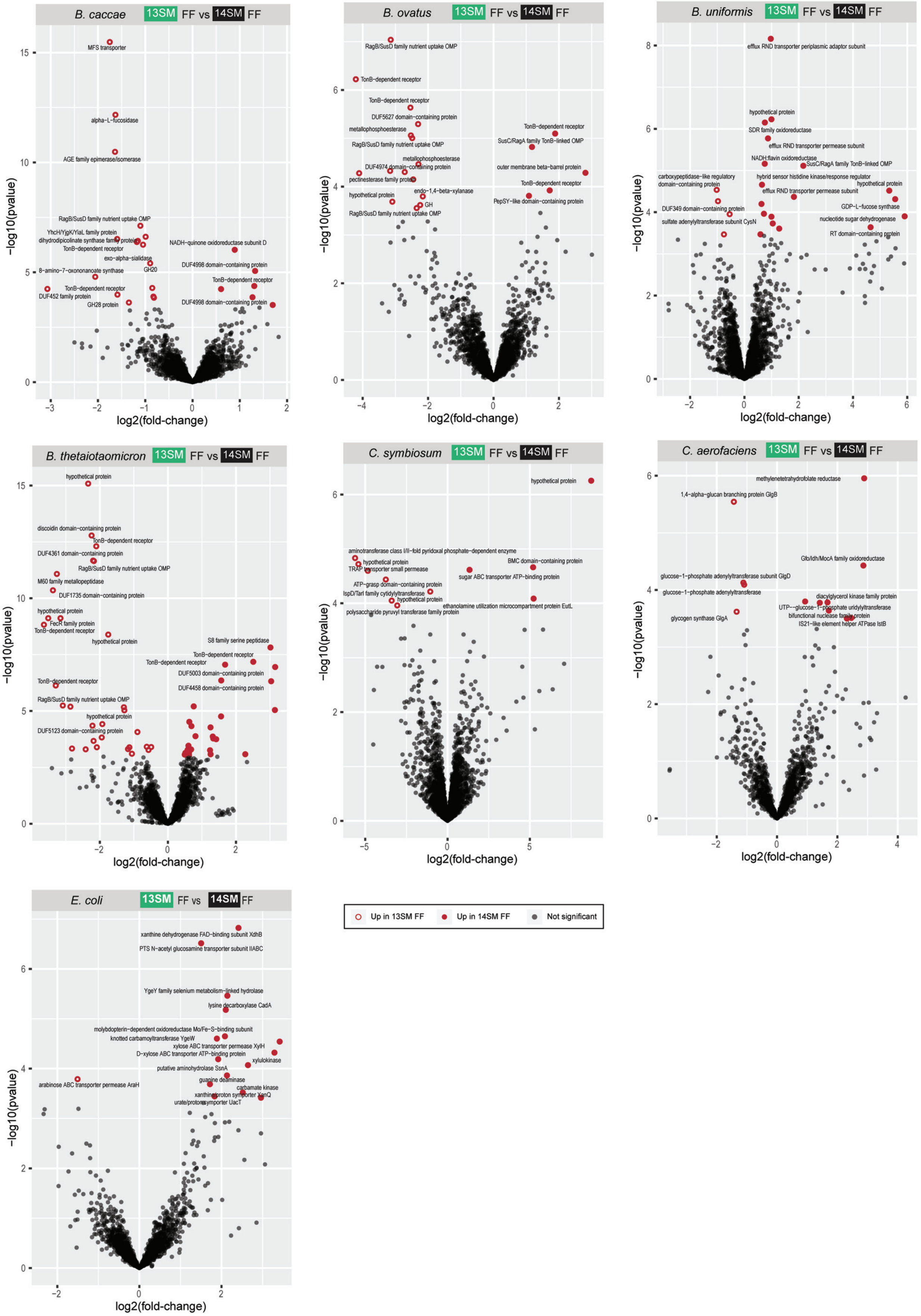
B. caccae 13SM FF vs 14SM FF
MFS transporter
alpha−L−fucosidase
AGE family epimerase/isomerase
RagB/SusD family nutrient uptake OMP
YhcH/YjgK/YiaL family protein
dihydrodipicolinate synthase family protein
TonB−dependent receptor
exo−alpha−sialidase
NADH−quinone oxidoreductase subunit D
8−amino−7−oxononanoate synthase
GH20
DUF4998 domain−containing protein
TonB−dependent receptor
TonB−dependent receptor
DUF452 family protein
GH28 protein
DUF4998 domain−containing protein
−log10(pvalue)
log2(fold−change)
B. ovatus 13SM FF vs 14SM FF
RagB/SusD family nutrient uptake OMP
TonB−dependent receptor
TonB−dependent receptor
DUF5627 domain−containing protein
metallophosphoesterase
TonB−dependent receptor
RagB/SusD family nutrient uptake OMP
SusC/RagA family TonB−linked OMP
metallophosphoesterase
DUF4974 domain−containing protein
outer membrane beta−barrel protein
pectinesterase family protein
TonB−dependent receptor
endo−1,4−beta−xylanase
hypothetical protein
PepSY−like domain−containing protein
GH
RagB/SusD family nutrient uptake OMP
−log10(pvalue)
log2(fold−change)
B. uniformis 13SM FF vs 14SM FF
efflux RND transporter periplasmic adaptor subunit
hypothetical protein
SDR family oxidoreductase
efflux RND transporter permease subunit
NADH:flavin oxidoreductase
SusC/RagA family TonB−linked OMP
carboxypeptidase−like regulatory domain−containing protein
hybrid sensor histidine kinase/response regulator
hypothetical protein
efflux RND transporter permease subunit
GDP−L−fucose synthase
DUF349 domain−containing protein
sulfate adenylyltransferase subunit CysN
nucleotide sugar dehydrogenase
RT domain−containing protein
−log10(pvalue)
log2(fold−change)
B. thetaiotaomicron 13SM FF vs 14SM FF
hypothetical protein
discoidin domain−containing protein
TonB−dependent receptor
DUF4361 domain−containing protein
RagB/SusD family nutrient uptake OMP
M60 family metallopeptidase
DUF1735 domain−containing protein
hypothetical protein
FecR family protein
TonB−dependent receptor
hypothetical protein
S8 family serine peptidase
TonB−dependent receptor
TonB−dependent receptor
DUF5003 domain−containing protein
DUF4458 domain−containing protein
TonB−dependent receptor
RagB/SusD family nutrient uptake OMP
hypothetical protein
DUF5123 domain−containing protein
−log10(pvalue)
log2(fold−change)
C. symbiosum 13SM FF vs 14SM FF
hypothetical protein
aminotransferase class I/II−fold pyridoxal phosphate−dependent enzyme
hypothetical protein
BMC domain−containing protein
TRAP transporter small permease
sugar ABC transporter ATP−binding protein
ATP−grasp domain−containing protein
IspD/TarI family cytidylyltransferase
hypothetical protein
ethanolamine utilization microcompartment protein EutL
polysaccharide pyruvyl transferase family protein
−log10(pvalue)
log2(fold−change)
C. aerofaciens 13SM FF vs 14SM FF
methylenetetrahydrofolate reductase
1,4−alpha−glucan branching protein GlgB
Gfo/Idh/MocA family oxidoreductase
glucose−1−phosphate adenylyltransferase subunit GlgD
glucose−1−phosphate adenylyltransferase
diacylglycerol kinase family protein
UTP−−glucose−1−phosphate uridylyltransferase
glycogen synthase GlgA
bifunctional nuclease family protein
IS21−like element helper ATPase IstB
−log10(pvalue)
log2(fold−change)
E. coli 13SM FF vs 14SM FF
xanthine dehydrogenase FAD−binding subunit XdhB
PTS N−acetyl glucosamine transporter subunit IIABC
YgeY family selenium metabolism−linked hydrolase
lysine decarboxylase CadA
molybdopterin−dependent oxidoreductase Mo/Fe−S−binding subunit
knotted carbamoyltransferase YgeW
xylose ABC transporter permease XylH
D−xylose ABC transporter ATP−binding protein
xylulokinase
putative aminohydrolase SsnA
arabinose ABC transporter permease AraH
guanine deaminase
carbamate kinase
xanthine/proton symporter XanQ
urate/proton symporter UacT
−log10(pvalue)
log2(fold−change)
Up in 13SM FF
Up in 14SM FF
Not significant

Figure EV5. Bacterial transcriptomes altered by *A. muciniphila* presence in fiber-deprived mice.

Volcano plots of synthetic microbiota members with >10 DEGs in the 13SM FF vs 14SM FF contrast (*B. caccae*, *B. ovatus*, *B. thetaiotaomicron*, *B. uniformis*, *C. symbiosum*, *C. aerofaciens*, and *E. coli*). RNA was extracted from cecal contents at 3 DPI in 13SM FF (*C. rodentium* resistant) and 14SM FF (*C. rodentium* susceptible) mice and individually mapped to the genome specified in Salmon. Features were filtered out if <2.5 cpm in four samples. Significance based on adjusted *p* value <0.05 based on Wald test adjusted using the Benjamini–Hochberg method in DESeq2. GH glycoside hydrolase, OMP outer membrane protein, RT reverse transcriptase.

 © The Author(s)
